# Factors Influencing Pediatric Nurses’ Infection Control Practices: A Cross-Sectional Study of Standard Precaution Knowledge, Self-Efficacy, and Organizational Culture

**DOI:** 10.3390/healthcare13111261

**Published:** 2025-05-27

**Authors:** Dasom Kim, Gaeun Kim

**Affiliations:** 1 Dongsan Hospital, Keimyung University, Daegu 42601, Republic of Korea; 2 College of Nursing, Keimyung University, Daegu 42601, Republic of Korea

**Keywords:** infection control, knowledge, self-efficacy, organizational culture, pediatric nursing

## Abstract

**Background:** Infection control is a critical component of pediatric nursing, as the patient population is highly vulnerable to healthcare-associated infections. This study aimed to evaluate the influence of standard precaution knowledge, self-efficacy, organizational culture, and environmental factors on infection control practices among pediatric nurses in South Korea. **Methods**: A descriptive, cross-sectional survey was conducted with 124 pediatric nurses employed in various hospitals. Data were analyzed using ANOVA and multiple regression to explore the relationships between infection control practices and the identified factors. **Results**: Infection control practices significantly varied by age (F = 4.95, *p* = 0.001) and clinical unit type (F = 3.27, *p* = 0.024). Positive correlations were observed between infection control practices and knowledge (r = 0.33), self-efficacy (r = 0.31), organizational culture (r = 0.34), and environmental factors (r = 0.35). Multiple regression analysis identified knowledge (β = 0.31), self-efficacy (β = 0.28), and organizational culture (β = 0.23) as significant predictors of infection control practices, collectively explaining 27% of the variance. **Conclusions**: Improving standard precaution knowledge and cultivating a supportive organizational culture are critical strategies to enhance pediatric nurses’ adherence to infection control practices. Interventions targeting these factors may significantly reduce infection risks in pediatric care settings.

## 1. Introduction

Healthcare-associated infections (HAIs) are a critical issue in healthcare settings, particularly for pediatric patients with immature immune systems. HAIs not only extend hospitalization and increase healthcare costs but also contribute significantly to morbidity and mortality rates [1]. Pediatric intensive care units (PICUs) face a risk of HAIs approximately five times higher than that of adult patients in general wards, primarily due to their weaker immune defenses and increased susceptibility to pathogen transmission [2,3]. Therefore, for pediatric nurses, providing high-quality care and implementing effective infection prevention measures are essential responsibilities [4].

As healthcare providers with the most frequent direct contact with patients, nurses are pivotal in preventing and managing infections. Standard precaution knowledge and competencies are vital in lowering infection rates within hospitals [5,6], and these skills are integral to the provision of safe, high-quality nursing care [6]. Despite this importance, research focusing specifically on standard precaution knowledge and practices among pediatric nurses is limited, and studies identifying factors influencing these practices are particularly scarce [6].

A solid foundation of knowledge is necessary for effective infection control, enabling nurses to implement preventive interventions in patient care [4]. According to the CDC, knowledge is a key antecedent to behavior, influencing the efficacy of infection prevention strategies when applied in clinical practice [7,8]. When nurses possess and apply standard precaution knowledge, they can significantly improve HAI prevention [9]. Self-efficacy, a concept in Bandura’s social cognitive theory, refers to the belief in one’s capability to perform necessary actions successfully [10]. Research by Mohammed et al. [11] emphasizes the role of self-efficacy in adherence to standard precautionary guidelines, underscoring the need for further investigation into its impact on infection control practices [12].

Organizational culture is another critical factor in infection control. Managerial support, team encouragement, effective communication, and feedback can foster adherence to infection prevention protocols [5]. Positive organizational culture is associated with a reduction in surgical site infections, highlighting the importance of a supportive environment in infection control [7]. Adequate staffing levels and the availability of safety equipment further support adherence to infection prevention guidelines [13].

The infection–prevention environment itself plays a pivotal role, providing healthcare staff with facilities, protective equipment, and administrative support necessary for safeguarding against infection risks during patient care [14]. The Korea Disease Control and Prevention Agency emphasizes the need for a safe healthcare environment, recommending sufficient resource allocation for infection prevention to protect both patients and healthcare providers [15,16].

This study was based on a multidimensional conceptual framework incorporating individual factors (standard precaution knowledge, self-efficacy) and organizational factors (organizational culture, infection–prevention environment) to explain infection control practices among pediatric nurses. These variables were selected based on theoretical relevance and evidence from previous studies on infection prevention behavior [10,12].

This study aims to explore the impact of standard precaution knowledge, self-efficacy, organizational culture, and the infection–prevention environment on infection control practices among pediatric nurses. The study focuses on assessing the levels of these factors and examining how they vary based on general characteristics. It also investigates the relationships between infection control practices and these variables, identifying the key factors that significantly influence infection control practices in this context.

## 2. Methods

### 2.1. Study Design

This study is a descriptive study that investigates the impact of standard precaution knowledge, self-efficacy, organizational culture, and the infection–prevention environment on infection control practices among pediatric nurses.

### 2.2. Participants

This study employed a convenience sampling method to recruit pediatric nurses from tertiary, general, and community hospitals located in D city. The study population consisted of registered nurses working in neonatal intensive care units (NICUs), general newborn nurseries (neonatal units), PICUs, and pediatric wards.

Inclusion criteria were as follows: (1) nurses currently working in one of the above-mentioned pediatric units, and (2) having at least one month of clinical experience in their current department. Exclusion criteria included nurses working in labor and delivery rooms, postpartum units, or obstetric wards, as well as those in administrative roles not directly involved in patient care.

The required sample size was calculated using G*Power 3.1.9.4 software, with a significance level of 0.05, a medium effect size of 0.15, and a power of 0.80, accounting for nine predictors (infection control practices, standard precaution knowledge, self-efficacy, organizational culture, and infection–prevention environment). Based on these parameters, the minimum sample size needed was determined to be 114 participants, with a target of 143 participants to account for a potential 20% attrition rate.

### 2.3. Research Instruments

1. Infection Control Practices

Infection control practices were evaluated using a modified instrument derived from the standard precaution guidelines of the U.S. Centers for Disease Control and Prevention (CDC), translated and subsequently adapted by Kim [17] to align with Korean standards for healthcare-associated infection precautions. This tool consists of 40 items, each rated on a 5-point Likert scale from 1 (“never practiced”) to 5 (“always practiced”), with higher scores indicating greater adherence to standard precautions. In Kim’s study [17], the instrument demonstrated high reliability with a Cronbach’s α of 0.91, while this study obtained a Cronbach’s α of 0.96.

2. Predisposing, Reinforcing, and Enabling Factors

Predisposing factors, which include personal motivations or justifications for performing a behavior, were assessed through measures of standard precaution knowledge and self-efficacy. Reinforcing factors, based on social learning theory, were evaluated by assessing organizational culture [18,19]. Enabling factors, which influence desirable behaviors and environmental change, were measured by assessing the infection–prevention environment.

(1) Standard Precaution Knowledge

Standard precaution knowledge was assessed using a tool originally developed by Cho [18] for ICU and emergency unit nurses, later refined by Baek [20] according to CDC guidelines. The instrument comprises 29 items related to standard precautions. Respondents answered each item with “Yes”, “No”, or “Don’t know”. Depending on the content, the correct answer could be either “Yes” or “No”, and scoring was based solely on the accuracy of the response. Each correct answer received 1 point, while incorrect answers and “Don’t know” responses received 0 points, yielding a total score range of 0 to 29. No reverse scoring procedure was applied, as all items were scored uniformly based on correctness rather than response direction. Higher total scores indicated greater knowledge of infection control. The reliability of the tool in this study was Cronbach’s α = 0.81. Content validity was established through expert panel review at the time of development. The instrument has been used in multiple studies involving Korean clinical nurses, supporting its contextual appropriateness and relevance to infection control assessment.

(2) Self-Efficacy

Self-efficacy was measured using a tool developed by Sherer et al. [21] and later modified by Kim [22] to align with standard precaution guidelines. Although the original self-efficacy scale developed by Sherer et al. [21] included several reverse-scored items, the version adapted and modified by Kim [22] for standard precaution compliance does not contain any reverse-scored items. All 22 items in the instrument are positively worded and were scored in the same direction, with higher scores indicating greater self-efficacy. This modification ensured scoring consistency and eliminated the need for reverse coding during analysis. The instrument includes 22 items, scored on a 5-point Likert scale from 1 (“strongly disagree”) to 5 (“strongly agree”), with higher scores representing greater self-efficacy. Previous reliability testing in Kim’s study [22] yielded a Cronbach’s α of 0.95, and in this study, Cronbach’s α was 0.98. Construct validity was supported by prior research using factor analysis and correlations with infection control behavior. The scale is grounded in social cognitive theory, which supports its relevance in predicting clinical nursing behavior.

Although both instruments contain multiple sub-domains, only total scores were calculated and used in the analysis. This decision followed the recommendations of the original tool developers and subsequent Korean adaptations, which interpret the scales as unified constructs with high internal consistency. Therefore, subscale-level analysis was not conducted in this study.

(3) Organizational Culture

The assessment of infection control organizational culture was based on the AHRQ organizational culture measurement tool [23], which was translated by Kim et al. [24] and further refined by Moon [25] to suit infection control guidelines in the Korean context. This 10-item instrument employs a 7-point Likert scale ranging from 1 (“strongly disagree”) to 7 (“strongly agree”), with higher scores reflecting a more positive organizational culture for infection control. The tool’s reliability was originally Cronbach’s α = 0.78 in the AHRQ study and Cronbach’s α = 0.85 in Moon’s [25] adaptation, while reliability in this study was Cronbach’s α = 0.92.

(4) Infection–Prevention Environment

The infection–prevention environment was assessed using a modified version of the defensive environment measurement tool developed by Han [26] and later adapted by An et al. [27] for nurses. This tool includes 11 items rated on a 5-point Likert scale from 1 (“strongly disagree”) to 5 (“strongly agree”), with higher scores indicating a more favorable infection–prevention environment. The original reliability was Cronbach’s α = 0.89, while An et al. [27] reported a Cronbach’s α of 0.85, and this study found a Cronbach’s α of 0.86.

### 2.4. Data Collection

Data collection was conducted between 31 March and 14 April 2022, at three tertiary hospitals, one general hospital, and one community hospital. At one tertiary hospital, the researcher visited the nursing unit to explain the study purpose and distribute surveys in person. For the remaining hospitals, the researcher contacted the nursing unit by phone to explain the study and distributed surveys by mail. Participating nurses completed the surveys anonymously, with each cover page detailing assurances of confidentiality, voluntary participation, and the right to withdraw at any point. Each survey took approximately 30 min to complete. To express gratitude, a small gift was provided to each participant. Out of 143 surveys distributed, 131 were returned, with 124 valid responses included in the final analysis after excluding seven incomplete surveys.

### 2.5. Data Analysis

Data were analyzed using IBM SPSS Statistics for Windows, version 25.0 (IBM Corp., Armonk, NY, USA). Descriptive statistics, including frequencies, percentages, means, and standard deviations, were used to summarize the demographic characteristics of participants and the levels of main study variables: infection control practices, standard precaution knowledge, self-efficacy, organizational culture, and the infection–prevention environment.

To examine differences in these variables according to general characteristics (e.g., age, highest academic degree, clinical experience, hospital type), independent sample t-tests were used for two-group comparisons, and one-way ANOVA was used for comparisons involving three or more groups. When significant group differences were observed in the ANOVA, Scheffé’s post hoc test was applied to determine the specific group differences. Pearson’s correlation coefficients were calculated to assess the bivariate relationships between infection control practices and the independent variables. To identify factors significantly predicting infection control practices, a stepwise multiple linear regression analysis was conducted. Before regression analysis, multicollinearity was examined using tolerance and variance inflation factor (VIF) values. Additionally, assumptions of linearity, normality, and homoscedasticity were tested through residual analysis and Cook’s distance to ensure model appropriateness. A *p*-value of less than 0.05 was considered statistically significant for all tests.

### 2.6. Ethical Considerations

This study was approved by the Institutional Review Board of Keimyung University (IRB No: 40525-202002-HR-089-03). All methods were carried out in accordance with relevant guidelines and regulations. All participants were informed of the study’s objectives, procedures, confidentiality measures, rights as participants, and data protection protocols. Written informed consent was obtained from each participant, and they were assured of their right to withdraw from the study at any time without consequence.

## 3. Results

### 3.1. General Characteristics of Participants

The mean age of participants was 28.95 ± 6.68 years. The majority held a bachelor’s degree as their highest academic degree (88.7%) and had over five years of total clinical experience (41.1%). Most participants (35.5%) had less than one year of experience in their current unit, and 96.8% were in a staff nurse position. A majority of nurses were employed at tertiary hospitals (81.5%), with the largest group working in general pediatric care units (49.1%).

Significant differences in infection control practices were observed based on age (F = 4.95, *p* = 0.001) and type of pediatric care unit (F = 3.27, *p* = 0.024). Nurses aged 31 years and older demonstrated higher levels of infection control practices than those in their twenties. Additionally, nurses working in NICUs and nurseries reported higher infection control practice scores than those in general wards. Organizational culture scores showed significant differences by highest academic degree (F = 4.18, *p* = 0.018), total clinical experience (F = 3.48, *p* = 0.018), experience in current unit (F = 2.79, *p* = 0.043), and hospital type (F = 4.12, *p* = 0.019). Infection–prevention environment scores significantly differed by highest academic degree (F = 3.60, *p* = 0.030) and hospital type (F = 3.30, *p* = 0.040) (Table 1).

### 3.2. Levels of Infection Control Practices, Standard Precaution Knowledge, Self-Efficacy, Organizational Culture, and Infection–Prevention Environment

The mean score for infection control practices was 4.51 (±0.42) out of a possible 5 points. Standard precaution knowledge scored a mean of 0.87 (±0.09) out of 1, while self-efficacy had a mean score of 4.17 (±0.59) out of 5. Organizational culture scored an average of 5.37 (±1.23) out of 7, and the infection–prevention environment had an average score of 4.35 (±0.57) out of 5 (Table 2).

### 3.3. Correlations Among Infection Control Practices, Standard Precaution Knowledge, Self-Efficacy, Organizational Culture, and Infection–Prevention Environment

Infection control practices showed significant positive correlations with standard precaution knowledge (r = 0.33, *p* < 0.001), self-efficacy (r = 0.31, *p* < 0.001), organizational culture (r = 0.34, *p* < 0.001), and infection–prevention environment (r = 0.35, *p* < 0.001). However, standard precaution knowledge did not significantly correlate with self-efficacy (r = −0.05, *p* = 0.584), organizational culture (r = 0.16, *p* = 0.080), or infection–prevention environment (r = 0.18, *p* = 0.050). Self-efficacy was significantly positively correlated with organizational culture (r = 0.21, *p* = 0.020) and the infection–prevention environment (r = 0.34, *p* < 0.001). Additionally, organizational culture showed a positive correlation with the infection–prevention environment (r = 0.44, *p* < 0.001) (Table 3).

### 3.4. Predictors of Infection Control Practices

Prior to conducting the regression analysis, multicollinearity was assessed. Due to a high correlation between age and total clinical experience, age was excluded from the model to reduce redundancy. Tolerance values ranged from 0.72 to 0.98, and variance inflation factor (VIF) values ranged from 1.01 to 1.38, indicating no multicollinearity concerns.

The assumptions of normality, linearity, and homoscedasticity were confirmed through residual analysis. Standardized residuals ranged from –2.81 to 2.54, and no outliers were detected, as all Cook’s distance values were below 1.

Stepwise multiple regression analysis was performed to identify factors associated with infection control practices. The overall model was statistically significant (F = 14.74, *p* < 0.001) and explained 27% of the variance in infection control practices among pediatric nurses (adjusted *R*^2^ = 0.27).

Three variables emerged as significant predictors of infection control practices among pediatric nurses. Specifically, standard precaution knowledge (β = 0.31, *p* < 0.001), self-efficacy (β = 0.28, *p* = 0.001), and organizational culture (β = 0.23, *p* = 0.006) were found to have a statistically significant positive association with infection control practices (Table 4).

## 4. Discussion

This study was guided by a conceptual framework that integrates both individual factors (standard precaution knowledge, self-efficacy) and organizational factors (organizational culture, infection–prevention environment) to explain infection control behaviors. The findings confirmed the utility of this framework, as it successfully identified key predictors of infection control practices. This framework may serve as a useful theoretical basis for designing future interventions focused on education and organizational support for infection prevention.

This study comprehensively analyzed factors affecting infection control practices among pediatric nurses, particularly focusing on standard precaution knowledge, self-efficacy, organizational culture, and the infection–prevention environment. Results indicated that standard precaution knowledge, self-efficacy, and organizational culture significantly influenced pediatric nurses’ infection control practices, collectively explaining 27% of the variance. These findings underscore the importance of enhancing standard precaution knowledge, strengthening self-efficacy, and fostering a positive organizational culture to improve infection control practices among pediatric nurses.

This study examined how individual and organizational factors—including standard precaution knowledge, self-efficacy, organizational culture, and the infection–prevention environment—affect infection control practices among pediatric nurses. These factors were selected to provide a multidimensional understanding of behavioral influences and support the development of targeted interventions. The study found that the average score for infection control practices among pediatric nurses was 4.51, higher than the 4.31 score reported in a previous study of emergency unit nurses [27]. The overall high level of infection control practices observed among pediatric nurses may reflect a heightened awareness of infection prevention due to the spread of emerging infectious diseases, such as COVID-19, and increased national-level monitoring and education efforts following previous infection control incidents, including the 2017 neonatal intensive care unit contamination case. These findings indicate that heightened awareness and regulatory reinforcement can contribute to improved infection control practices.

Among the factors influencing infection control practices, standard precaution knowledge emerged as a crucial factor, aligning with previous studies that underscore the necessity of standard precaution knowledge for effective infection control practices [14,15]. The standard precaution knowledge serves as an antecedent to health behavior, enabling nurses to perform effective preventive actions against infections [6]. Given the high error rates in certain knowledge items, there is a clear need for regular, targeted training to ensure pediatric nurses possess accurate, up-to-date standard precaution knowledge for infection control.

Self-efficacy was identified as the second key factor influencing infection control practices. Higher self-efficacy was associated with more rigorous adherence to infection control guidelines, echoing findings from previous studies that highlight the positive impact of self-efficacy on infection control behaviors [14,28]. Self-efficacy reflects a nurse’s belief in their ability to perform infection control tasks effectively, and its reinforcement can enhance adherence to infection protocols. Studies have shown that infection control training positively impacts self-efficacy among nursing students and practicing nurses, suggesting that training programs should focus on boosting self-efficacy to improve infection control practices [22,29].

These findings suggest that infection control performance is not solely dependent on individual knowledge, but is also significantly shaped by internal motivation and the perceived support from the workplace environment. In particular, self-efficacy may serve as a mediating factor between knowledge and actual behavior, which aligns with Bandura’s social cognitive theory. Furthermore, the influence of organizational culture indicates that institutional norms and leadership can either facilitate or hinder the translation of knowledge into consistent practice. These results emphasize the need for organizational-level interventions—not just individual education—to sustain effective infection control behaviors among pediatric nurses.

Additionally, the study confirmed that organizational culture significantly impacts pediatric nurses’ infection control practices. Previous research with emergency and hospital nurses has similarly identified organizational culture as a critical factor influencing infection control practices [7,27,30]. A positive organizational culture fosters infection control practices by encouraging norms, behaviors, and collaboration among staff members [6]. Within a supportive organizational culture, team members adhere to shared infection control norms and motivate each other to uphold standards [25,27]. This suggests that organizational support and resources are essential for promoting infection control, underscoring the importance of cultivating a positive organizational culture to encourage adherence to infection control practices.

While the infection–prevention environment was positively correlated with infection control practices, it did not emerge as a significant predictor. This differs from previous studies [27] and may indicate that environmental factors primarily provide structural support for infection control rather than directly influencing nurse behavior. Effective infection control practices may require a supportive organizational culture to complement the physical resources provided by the environment. The Korean Society of Healthcare-Associated Infection Control and Prevention (2017) highlights the necessity of creating a safe healthcare environment and providing sufficient resources for infection prevention [15], suggesting that the interaction between organizational culture and environmental support may be critical in driving effective infection control practices.

Despite its strengths, this study has limitations. First, the study was conducted with a limited sample of pediatric nurses from hospitals in D city, which may restrict the generalizability of the findings. Further studies should consider broader and more diverse settings to confirm these results. Second, the study relied on self-reported surveys to assess infection control practices, which may not accurately reflect actual behaviors due to social desirability bias. Future research should incorporate observational methods alongside self-reported data to compare and verify infection control practices.

Previous studies on infection control practices have primarily targeted adult patient care settings, with a notable lack of focus on pediatric nurses despite their unique clinical environments and infection risks. Additionally, many earlier studies explored only one or two influencing factors, such as knowledge or attitudes, without incorporating organizational aspects such as culture or systemic support. This study addresses these limitations by applying a multidimensional approach that integrates both individual and organizational predictors. By doing so, it provides a more comprehensive understanding of infection control practices in pediatric nursing and offers practical implications for training and institutional policy.

This study makes a distinct contribution to the field of infection control by focusing specifically on pediatric nurses—a population that has received limited attention in previous research. While many prior studies have concentrated on nurses in adult wards, intensive care units, or emergency departments, this study addresses a significant gap by exploring factors influencing infection control practices in pediatric care settings. Additionally, this research employed a multidimensional framework that integrates both individual factors (standard precaution knowledge and self-efficacy) and organizational factors (organizational culture and infection–prevention environment), offering a more comprehensive analysis than previous studies that focused on isolated variables. By identifying standard precaution knowledge, self-efficacy, and organizational culture as significant predictors, the study provides valuable insights for developing targeted and holistic interventions to enhance infection control practices in pediatric nursing.

Nonetheless, this study offers valuable insights into the factors influencing infection control practices among pediatric nurses, particularly in the context of heightened awareness following the COVID-19 pandemic. Unlike previous studies primarily focused on nurses in adult wards, intensive care, and emergency settings, this study highlights the unique infection control needs of pediatric nurses. These findings provide a foundational basis for developing infection control programs tailored to pediatric nursing, contributing important insights to the field.

This study contributes to the limited literature on pediatric nurses by addressing infection control through both individual and organizational factors. It provides a comprehensive framework that emphasizes the role of self-efficacy and organizational culture in shaping infection control behaviors.

Future research should explore causal relationships using longitudinal or experimental designs. Qualitative studies may offer deeper insights, and broader sampling across various regions and pediatric care settings could improve generalizability.

## 5. Conclusions

This study aimed to investigate infection control practices, standard precaution knowledge, self-efficacy, organizational culture, and infection–prevention environment among pediatric nurses, identifying correlations and the factors that significantly influence infection control practices. The findings revealed that standard precaution knowledge, self-efficacy, and organizational culture were significant predictors of infection control practices, collectively explaining 27% of the variance. To enhance infection control practices among pediatric nurses, it is essential to provide detailed and practical training that considers the unique aspects of pediatric care, thereby strengthening self-efficacy. Additionally, fostering a supportive organizational culture that encourages open communication, regular feedback, and monitoring among nursing staff and administrators can further reinforce adherence to standard precaution protocols.

This study, while informative, relied on self-reported questionnaires, which may limit the objectivity of the findings. Future research should incorporate direct observational methods to evaluate infection control practices more accurately. Furthermore, the development and assessment of targeted infection control programs specifically designed for pediatric nurses are recommended to build on these findings, ultimately supporting better infection control outcomes in pediatric care settings.

## Figures and Tables

**Table 1:** Differences in variables according to participants’ characteristics.

								(n = 124)					
Characteristics	Categories	n (%)	M ± SD	Infection Control Practices		Standard Precaution Knowledge		Self-Efficacy		Organizational Culture		Infection–Prevention Environment	
				M ± SD	t or F (*p*)	M ± SD	t or F (*p*)	M ± SD	t or F (*p*)	M ± SD	t or F (*p*)	M ± SD	t or F (*p*)
Age (year)	<24	25 (20.2)	28.95 ± 6.68	4.44 ± 0.44	4.95 (0.010)	0.84 ± 0.11	2.62 (0.082)	4.13 ± 0.53	0.69 (0.599)	5.16 ± 1.26	2.30 (0.064)	4.27 ± 0.57	1.29 (0.280)
	25~29	70 (56.4)		4.44 ± 0.31		0.90 ± 0.04		4.23 ± 0.66		5.13 ± 1.40		4.31 ± 0.62	
	≥30	29 (23.4)		4.79 ± 0.27		0.90 ± 0.06		4.31 ± 0.58		5.96 ± 1.08		4.56 ± 0.55	
Highest academic degree	College	7 (5.6)		4.65 ± 0.37	1.97 (0.143)	0.90 ± 0.05	0.71 (0.494)	4.19 ± 0.39	1.34 (0.266)	6.21 ± 0.37	4.18 (0.018)	4.86 ± 0.10	3.60 (0.030)
	Bachelor	110 (88.8)		4.49 ± 0.43		0.87 ± 0.10		4.15 ± 0.60		5.26 ± 1.25		4.31 ± 0.57	
	≥Masters	7 (5.6)		4.79 ± 0.34		0.91 ± 0.03		4.53 ± 0.60		6.27 ± 0.68		4.55 ± 0.52	
Hospital type	Tertiary hospital	101 (81.5)		4.56 ± 0.38	2.82 (0.630)	0.88 ± 0.07	1.47 (0.232)	4.20 ± 0.56	0.48 (0.620)	5.47 ± 1.12 ^a^	4.12 (0.019)	4.39 ± 0.55 ^a^	3.30 (0.040)
	General hospital	15 (12.0)		4.37 ± 0.42		0.86 ± 0.11		4.13 ± 0.67		5.30 ± 1.49 ^a^		4.37 ± 0.56 ^a^	
	Clinics	8 (6.5)		4.28 ± 0.81		0.83 ± 0.22		4.00 ± 0.82		4.21 ± 1.56 ^b^		3.86 ± 0.72 ^b^	
Total clinical experience (year)	<1	8 (6.5)	5.98 ± 6.74	4.42 ± 0.40	2.48 (0.640)	0.85 ± 0.09	1.67 (0.176)	3.91 ± 0.67	2.46 (0.063)	4.71 ± 1.42 ^a^	3.48 (0.018)	3.92 ± 0.80	2.50 (0.063)
	1~2	35 (28.2)		4.44 ± 0.42		0.86 ± 0.10		4.21 ± 0.58		5.22 ± 1.17 ^b^		4.27 ± 0.50	
	3~4	30 (24.2)		4.28 ± 0.50		0.86 ± 0.12		3.98 ± 0.59		5.06 ± 1.32 ^b^		4.39 ± 0.60	
	≥5	51 (41.1)		4.64 ± 0.36		0.90 ± 0.06		4.30 ± 0.56		5.76 ± 1.10 ^c^		4.46 ± 0.53	
Experience in current unit (year)	<1	44 (35.5)	2.85 ± 2.58	4.43 ± 0.47	1.61 (0.191)	0.87 ± 0.11	1.21 (0.310)	4.11 ± 0.59	0.61 (0.610)	5.06 ± 1.23 ^a^	2.79 (0.043)	4.30 ± 0.56	1.24 (0.297)
	1~2	28 (22.6)		4.54 ± 0.40		0.85 ± 0.12		4.27 ± 0.59		5.36 ± 1.24 ^a^		4.23 ± 0.63	
	3~4	31 (25.0)		4.52 ± 0.43		0.89 ± 0.06		4.13 ± 0.63		5.41 ± 1.39 ^a^		4.47 ± 0.56	
	≥5	21 (16.9)		4.67 ± 0.30		0.89 ± 0.03		4.26 ± 0.54		5.98 ± 0.68 ^b^		4.46 ± 0.50	
Job position	General nurse	120 (96.8)		4.52 ± 0.42	2.72 (0.070)	0.87 ± 0.09	0.65 (0.524)	4.15 ± 0.60	1.35 (0.263)	5.32 ± 1.24	2.35 (0.100)	4.34 ± 0.58	0.39 (0.675)
	Charge nurse	4 (3.2)		4.83 ± 0.18		0.92 ± 0.04		4.47 ± 0.62		6.63 ± 0.56		4.55 ± 0.43	
Type of pediatric care unit	Ward	61 (49.1)		4.41 ± 0.45 ^a^	3.27 (0.024)	0.86 ± 0.11	1.14 (0.334)	4.11 ± 0.63	0.43 (0.734)	5.24 ± 1.30	1.70 (0.171)	4.29 ± 0.61	1.60 (0.194)
	NICU	40 (32.3)		4.56 ± 0.40 ^b^		0.87 ± 0.07		4.24 ± 0.53		5.27 ± 1.33		4.32 ± 0.53	
	PICU	13 (10.5)		4.68 ± 0.28 ^b^		0.90 ± 0.05		4.21 ± 0.64		5.90 ± 0.65		4.65 ± 0.37	
	Nursery	10 (8.1)		4.78 ± 0.30 ^b^		0.91 ± 0.04		4.24 ± 0.57		5.88 ± 0.72		4.46 ± 0.59	

Superscript letters (a–c) indicate statistically significant differences among groups based on post-hoc Scheffé test (*p* < 0.05). Groups not sharing the same letter differ significantly.

**Table 2:** Scores for the investigated variables.

				(n = 124)
Variables	Range	M ± SD	Min	Max
Infection control practices	1–5	4.51 ± 0.42	2.58	5.00
Standard precaution knowledge	0–1	0.87 ± 0.09	0.31	0.97
Self-efficacy	1–5	4.17 ± 0.59	2.91	5.00
Organizational culture	1–7	5.37 ± 1.23	1.50	7.00
Infection–prevention environment	1–5	4.35 ± 0.57	2.64	5.00

**Table 3:** Correlations between variables.

			(n = 124)		
	Infection Control Practices	Standard Precaution Knowledge	Self-Efficacy	Organizational Culture	Infection–Prevention Environment
	r (*p*)	r (*p*)	r (*p*)	r (*p*)	r (*p*)
Infection control practices	1				
Standard precaution knowledge	0.33 (<0.001)	1			
Self-efficacy	0.31 (<0.001)	−0.05 (0.584)	1		
Organizational culture	0.34 (<0.001)	0.16 (0.080)	0.21 (0.020)	1	
Infection–prevention environment	0.35 (<0.001)	0.18 (0.050)	0.34 (<0.001)	0.44 (<0.001)	1

**Table 4:** Predictors of infection control practices among pediatric nurses.

					(n = 124)
Variables	B	SE	β	t	*p*
(Constant)	2.01	0.40		5.00	<0.001
Standard precaution knowledge	1.43	0.36	0.31	3.93	<0.001
Self-efficacy	0.20	0.06	0.28	3.52	0.001
Organizational culture	0.08	0.03	0.23	2.82	0.006

*R*^2^ = 0.33, adjusted *R*^2^ = 0.27, F = 14.74, *p* < 0.001.

## Data Availability

All data generated or analyzed during this study are included in this published article.
